# Development of a manualized protocol of massage therapy for clinical trials in osteoarthritis

**DOI:** 10.1186/1745-6215-13-185

**Published:** 2012-10-04

**Authors:** Ather Ali, Janet Kahn, Lisa Rosenberger, Adam I Perlman

**Affiliations:** 1 Department of Pediatrics, Yale University School of Medicine, 2 Church Street South, New Haven, CT, 06519, USA; 2 College of Medicine, University of Vermont, 240 Maple Street, Burlington, VT, 05401, USA; 3 Yale-Griffin Prevention Research Center, Griffin Hospital, 130 Division Street, Derby, CT, 06418, USA; 4 Duke Integrative Medicine, Duke University School of Medicine, 3475 Erwin Road, Durham, NC, 27710, USA

**Keywords:** Massage, Manualization, Clinical trial, Manual therapy, Swedish

## Abstract

**Background:**

Clinical trial design of manual therapies may be especially challenging as techniques are often individualized and practitioner-dependent. This paper describes our methods in creating a standardized Swedish massage protocol tailored to subjects with osteoarthritis of the knee while respectful of the individualized nature of massage therapy, as well as implementation of this protocol in two randomized clinical trials.

**Methods:**

The manualization process involved a collaborative process between methodologic and clinical experts, with the explicit goals of creating a reproducible semi-structured protocol for massage therapy, while allowing some latitude for therapists’ clinical judgment and maintaining consistency with a prior pilot study.

**Results:**

The manualized protocol addressed identical specified body regions with distinct 30- and 60-min protocols, using standard Swedish strokes. Each protocol specifies the time allocated to each body region. The manualized 30- and 60-min protocols were implemented in a dual-site 24-week randomized dose-finding trial in patients with osteoarthritis of the knee, and is currently being implemented in a three-site 52-week efficacy trial of manualized Swedish massage therapy. In the dose-finding study, therapists adhered to the protocols and significant treatment effects were demonstrated.

**Conclusions:**

The massage protocol was manualized, using standard techniques, and made flexible for individual practitioner and subject needs. The protocol has been applied in two randomized clinical trials. This manualized Swedish massage protocol has real-world utility and can be readily utilized both in the research and clinical settings.

**Trial registration:**

Clinicaltrials.gov NCT00970008 (18 August 2009)

## Background

Among the challenges in research in complementary and alternative medicine (CAM) is the necessity to design clinical trials that are methodologically rigorous as well as consistent with prevailing clinical practice patterns [1-3]. This difficulty has been mentioned in trials of botanical medicines [3], mind-body interventions [4], and manual therapies [5,6]. Many CAM disciplines espouse patient-centered care that often results in some individualization of treatment. Thus, standardization of interventions for clinical trials poses a particular challenge. Clinical trial design of manual therapies may be especially challenging since techniques are often practitioner-dependent as well as patient-oriented [7,8].

Massage therapy is one of the most popular CAM techniques in the USA [9]. Between 2002 and 2007, the 1-year prevalence of use of massage by the US adult population increased from 5% (10 million) to 8.3% (18 million) [9]. Massage is generally used, with some research support, to relieve pain from musculoskeletal disorders and cancer, rehabilitate sports injuries, reduce stress, increase relaxation, decrease feelings of anxiety and depression, and aid in general wellness [10-24].

The identification of massage therapy for patients with osteoarthritis as a research priority derives directly from a CDC-funded systematic evidence mapping project applied to CAM [25], leading to our pilot study evaluating the safety and efficacy of a 1-h whole body Swedish massage in adults with osteoarthritis of the knee [26]. After 8 weeks of massage therapy (biweekly × 4 weeks, weekly × 4 weeks), scores on the Western Ontario and McMaster Universities Arthritis Index (WOMAC) [27] global score improved significantly (55%) from baseline (*P* <0.001), as did the scores in each subdomain (pain, stiffness, and physical functional disability), with effects persisting 8 weeks after treatment cessation [26]. Swedish massage techniques were chosen for their practical utility; being the most widely taught and practiced massage method that is well defined procedurally, and safe when administered by trained massage therapists [28-30].

Manualization was initially developed for the creation of standardized treatment protocols for psychotherapy, both to help provide methodologic rigor for evaluation, and as a means to provide specificity and guidelines regarding individualized treatment [31]. Massage therapy, as an intervention in clinical trials has similar needs for methodologic rigor to standardize patient-customized treatments and practitioner variation [32,33]. In 2002, Schnyer and Allen published their methodology on developing treatment manuals for acupuncture interventions used in NIH-funded trials. These manuals served as a means to facilitate the training process, enable evaluation of conformity and competence, and increase the ability to identify the active therapeutic ingredients in clinical trials of acupuncture [34]. The Institute of Medicine has noted that manualization is an integral component for rigorous research on CAM therapies [35].

This investigative team collaborated again in a 2-year single-blinded randomized controlled dose-finding study, aiming to identify an optimal-practical dose and treatment regimen of an 8-week course of Swedish massage for osteoarthritis of the knee. The intervention development phase of this dose-finding study incorporated a formal manualization process. Here we describe our approach in developing a standardized massage intervention tailored to subjects with osteoarthritis of the knee while respectful of the individualized nature of massage therapy [34], as well as the implementation of this manualized protocol into two NIH-supported randomized controlled trials.

## Methods

Development of the manualization process, conducted over the course of 2 months, involved the input of methodologic and clinical experts. The manualization process was aided by a committee organized and under the direction of the former Research Director (JK) of the Massage Therapy Research Consortium (MTRC). The MTRC was a consortium of schools in the United States and Canada collaborating to build research capacity and to advance research in the field of massage.

Four meetings were held by telephone conference call. Participants included members of the investigative team, massage therapists from both clinical sites, and massage therapy researchers. Each meeting reiterated the overarching goals of the manualization process:

1. To create a reproducible, semi-structured protocol for massage therapy for osteoarthritis of the knee, while allowing for some latitude based on therapists’ clinical judgment. Four distinct ‘doses’ varying on duration (30 min *vs*. 60 min) and frequency (weekly or biweekly) to assess dose–response effects.

2. To be consistent with the protocol delivered during the pilot study [26]. Thus, only the Swedish massage techniques of effleurage, petrissage, tapotement, vibration (including rocking or jostling), friction, and skin-rolling were to be used. These are standard Swedish strokes and massage techniques taught in schools accredited by the Commission on Massage Therapy Accreditation (COMTA) [36].

The manualization team tailored the treatment protocols to the over-arching goals of subsequent clinical trials; that is to determine the efficacy of a standardized Swedish massage protocol for treatment of patients with osteoarthritis of the knee. The putative mechanisms of massage as related to treating osteoarthritis (relaxation, reducing inflammation, improving flexibility) were considered when designing the protocol.

The protocol of the dose-finding study, consent form and all recruitment materials were approved by the Institutional Review Boards of the University of Medicine and Dentistry of New Jersey (Newark, NJ, USA), Griffin Hospital (Derby, CT, USA), and the Saint Barnabas Medical Center (Livingston, NJ, USA). The study was conducted in accordance with the Declaration of Helsinki [37].

## Results

The manualized protocol specifies the body regions to be addressed, with distinct 30- and 60-min protocols, as well as the standard Swedish strokes to be used (effleurage, petrissage, tapotement, vibration, friction, and skin rolling) [38] (see Table 1). Each protocol specifies the time allocated to various body regions (lower/upper limbs, lower/upper back, head, neck, chest) and specific areas of emphasis. The order of body regions, patient position (supine or prone), technique sequence, or technique type is left to the discretion of the therapist to account for individual practitioner preference and patient needs.

**Table 1:** 30- and 60-minute massage protocols

	**Allowed Swedish massage techniques:**	
	**Effleurage, petrissage, tapotement, vibration, friction, and skin rolling**	
	**30-minute protocol (25 minutes of table time)**	
**Region**	**Time allotted**	**Distribution**
Lower limbs	12 to 15 min	From knee down including lower leg, ankle, and foot. From knee up including hips, pelvis, buttocks, and thigh.
	(45% to 50% of session)	
Upper body	8 to 12 min	Lower and upper back; head/neck/chest.
	(36% to 44% of session)	
Discretionary	2 to 5 min	Therapist to expand treatment to other affected areas; that is rib cage, flank, upper limbs, et cetera.
	(6% to 19% of session)	
	**60-minute protocol (55 minutes of table time**^**a**^**)**	
Lower limbs	20 to 27.5 min	From knee down including lower leg, ankle, and foot. From knee up including hips, pelvis, buttocks, and thigh.
	(45% to 50% of session)	
Upper body	15 to 24 min	Lower and upper back; head, neck, and chest.
	(36% to 44% of session)	
Discretionary	3.5 to 20 min	Therapist to expand treatment to other affected areas; that is rib cage, flank, upper limbs, et cetera.
	(6% to 19% of session)	

^a^Accounting for time spent in transition including the welcome, transition to the massage room, taking off jewelry, and other preparatory activities.

The protocol further specifies intentions/attentions for the study therapists consistent with massage therapy practice, specifically:

1. Assess and address relevant imbalances in posture

2. In general, seek to establish symmetry

3. Strengthen muscles around knee joint

4. Compensate weak muscles

5. Disperse stress to bring balance

6. Decrease sympathetic activity

7. Diffuse inflammation

8. Reduce inhibition in anti-gravity muscles

Each study therapist was trained in the protocol, and signed a form attesting to adherence to the manualized massage protocol after each massage session. No deviations from the protocol were reported for the duration of the dose-finding trial at either site.

The manualization team agreed that the knee must be regarded as a functional unit. Thus, the protocol explicitly does not specify the percent of time to be spent directly on structures of the knee. Rather, time variables included the upper and lower leg, both including the knee (see Table 1).

The manualized 30- and 60-min protocols were implemented in a 24-week randomized dose-finding trial of massage therapy for osteoarthritis of the knee [39]. Subjects (*n*=125) were randomized to one of four regimens of the manualized massage intervention (30 min or 60 min weekly or biweekly) or to a usual care control. Outcomes were assessed at baseline, 8, 16, and 24 weeks and included the WOMAC, visual analog pain scale, range of motion, and time to walk 50 feet. The initial randomization occurred in October 2009 and the last subject completed the 8-week intervention in October 2010.

Both 60-min regimens (weekly or biweekly) demonstrated significantly improved WOMAC global scores (24.0 points, 95% CI varied from 15.3 to 32.7) compared to usual care (6.3 points, 95% CI 0.1 to 12.8) at the primary endpoint of 8 weeks. Further, the 60-min regimens demonstrated significant improvements in WOMAC subscales of pain and functionality, as well as the visual analog pain scale compared to usual care. No significant differences were seen in range of motion at 8 weeks, and no significant effects were seen in any outcome measure at 24 weeks compared to usual care. A dose–response curve based on WOMAC global scores shows increasing effect with greater total time of massage; with 60-min doses scoring significantly better than 30-min doses. No significant differences were seen in WOMAC global scores between the 60-min doses (weekly or biweekly) [39].

This trial thus established an ‘optimal-practical’ dose (60-min once-weekly) of this manualized Swedish massage regimen for osteoarthritis of the knee. This decision was based on the superiority of the 60-min compared to 30-min regimens, the essentially similar outcomes of the two 60-min doses, the convenience of a once-weekly protocol (compared to biweekly), cost savings, and consistency with a typical real-world massage protocol [39].

This optimized dose of manualized Swedish massage therapy is currently being implemented in a large-scale (*n*=222) NIH-funded 52-week efficacy trial of massage therapy for osteoarthritis of the knee at three clinical sites [40].

## Discussion

Clinical trials of massage therapy are inherently challenged by an inability to blind practitioner and recipient. Furthermore, massage practices are heterogeneous with procedures utilized from different schools of massage incorporating a variety of techniques. Some of these schools include Swedish massage, neuromuscular, myofascial, Chinese, other Asian, medical, osteopathic, or naturopathic manipulative therapies [41].

Massage is a pleasant and desirable intervention and is safe when delivered by trained practitioners using standard Swedish techniques [28]. Demonstrating the efficacy of massage therapy in clinical trials requires reproducible treatment regimens.

To our knowledge, this is the first report to describe the manualization of massage therapy. The feasibility of this protocol is demonstrated by implementing this standardized regimen in two clinical sites in the randomized dose-finding trial [39], as well as in a larger three-site efficacy trial [40].

A few published reports of implementing standardized Swedish massage regimens in randomized trials exist. Patterson *et al.* published a standardized massage (and control) regimen in a clinical trial assessing fatigue reduction in cancer chemotherapy, though no results have been published [36]. Sharpe *et al.* published the results of a pilot randomized trial assessing the effects of a standardized Swedish massage regimen *vs.* guided relaxation on stress and wellbeing in a pilot study (*n*=54) [42], though there are no reports of implementing this regimen in a larger sample. Taylor *et al.* also report using a standardized Swedish massage protocol though the protocol was not described to the point that the intervention could be reproduced [43]. Cherkin *et al.* assessed a standardized Swedish protocol (‘relaxation massage’) [44], other massage techniques (‘structural massage’), and continuing usual care in a three-arm randomized controlled trial for patients with chronic back pain. Both massage regimens were found to be superior to usual care, with no clinically meaningful differences seen between the relaxation and structural massage arms [7].

Other randomized trials of Swedish massage therapy have not used standardized massage interventions, compromising external validity and reducing the ability to replicate positive results [45,46].

The concept of ‘dose’ has never been formally defined for massage. Prior to determining specific study protocols, the manualization team had to operationally define ‘dose’ of massage therapy. If dose, for example, was defined only by the length of time, it may be assumed that a single 60-min session and two 30-min sessions would be equivalent. This assumption was tested by assessing the effects of frequency of massage therapy. Thus, in our manualization process, two variables germane to dosing were assessed: frequency of massage therapy and duration of treatment. Frequency was varied between weekly or biweekly sessions, based on practicality and current practice standards. Duration of treatment was negotiated by the expert panel to provide a dose that is clinically effective while avoiding possible overtreatment.

Finally the team explored the issue of what constituted ‘massage for the knee’. In this study the team chose to view the knee in its functionality and distribute the apportioned time not to specific muscles, tendons or ligaments, but rather to the two regions of the knee and lower leg (ankle, foot, and lower leg) and the knee and upper leg (including hips, pelvis, buttocks, and thighs).

Massage treatments are often focused on a particular functional issue or anatomic region, though they typically also include some broader treatment to promote relaxation [7]. Relaxation has been thought to be helpful to many healing processes, and from a massage therapy perspective, to aid in whole body integration to supporting proper gait and biomechanics of the joint(s). Thus, the protocol involved regions beyond the knee; time was allotted to the upper and lower back, neck, chest, and head.

One of the limitations of this manualized Swedish massage protocol is that the protocol may not be as efficacious as real-world practice as fully individualized treatment is precluded. In addition, other techniques (that is, neuromuscular and myofascial) may be more effective in altering posture and gait in ways that might affect osteoarthritis symptoms and progression. The one known study comparing Swedish massage with myofascial and neuromuscular techniques for treatment of back pain showed no significant difference in ability to affect pain or function [7].

## Conclusions

The resulting massage protocol was manualized [40], using standard Swedish techniques [26,28], and made flexible for individual subject variability. This manualized Swedish massage protocol has successfully been implemented in a dual-site dose-finding clinical trial and a three-site efficacy trial. The manualized Swedish massage protocol has real-world utility and can be readily utilized in clinical trials and clinical practice.

## Abbreviations

CAM: Complementary and Alternative Medicine; CDC: Centers for Disease Control and Prevention; MTRC: Massage Therapy Research Consortium; NCCAM: National Center for Complementary and Alternative Medicine; NIH: National Institutes of Health; WOMAC: Western Ontario and McMaster Universities Arthritis Index.

## Competing interests

The authors declare that they have no competing interests.

## Authors’ contributions

AA led the manualization process, participated in the design and coordination of the study, and drafted the manuscript. JK provided technical expertise in massage therapy. LR assisted in the clinical trial and provided critical review of the manuscript. AP conceived of the study, and participated in its design and coordination, and critically reviewed the manuscript. All authors read and approved the final manuscript.
